# Infiltration of CD1a-positive dendritic cells in advanced laryngeal cancer correlates with unfavorable outcomes post-laryngectomy

**DOI:** 10.1186/s12885-021-08715-6

**Published:** 2021-08-30

**Authors:** Akimichi Minesaki, Keita Kai, Yuichiro Kuratomi, Shinichi Aishima

**Affiliations:** 1 Department of Pathology & Microbiology, Saga University Faculty of Medicine, Saga, Japan; 2 Department of Otolaryngology – Head & Neck Surgery, Saga University Faculty of Medicine, Saga, Japan; 3 Department of Pathology, Saga University Hospital, Nabeshima 5-1-1, Saga City, Saga, 849-8501 Japan

**Keywords:** Laryngeal cancer, CD1a, Dendritic cell, Prognosis, Immunohistochemistry

## Abstract

**Background:**

The prognosis of advanced laryngeal cancer is unfavorable despite advances in multidisciplinary therapy. Dendritic cells (DCs) play a central role in antitumor immunity. Tumor-infiltrating CD1a^+^ DCs have been reported to be associated with clinical outcomes in carcinomas of various organs, but the clinical impact of CD1a^+^ DCs in laryngeal cancer remains to be unequivocally established.

**Methods:**

We retrospectively analyzed the cases of 57 patients with Stage III or IV laryngeal cancer who underwent a total laryngectomy. Immunohistochemistry detection of CD1a, S100 and CD8 was performed on representative resected specimens. CD1a^+^ DCs, S100^+^ DCs and CD8^+^ cytotoxic T-lymphocytes (CTLs) were evaluated, and the cases divided into high and low groups according to the cut-off of the median values for each of these 3 parameters.

**Results:**

Compared to the CD1a-low group, the CD1a-high group had more advanced cases and showed significantly worse disease-specific survival (DSS) (*P* = 0.008) and overall survival (OS) (*P* = 0.032). The analyses of S100 DCs and CD8^+^ CTLs revealed no significant impact on clinical outcomes. However, multivariate analysis revealed that infiltration of CD1a^+^ DCs was an independent unfavorable prognostic factor for both DSS (*P* = 0.009) and OS (*P* = 0.013).

**Conclusions:**

Our results demonstrated that the infiltration of CD1a^+^ DCs was associated with unfavorable clinical outcomes in patients with advanced laryngeal cancer who underwent a total laryngectomy as the initial treatment.

## Background

Laryngeal cancer is the second most common malignancy of head and neck cancer, with thyroid cancer being the first [1]. Although laryngeal cancer accounts for only 0.9% of cancers in men and 0.2% in women, circa 4800 patients are newly diagnosed with laryngeal cancer every year in Japan [2]. The 5-year survival rate of early laryngeal cancer (Stages I and II in the TNM classification) is favorable (> 80%), but the prognosis of advanced laryngeal cancer (Stages III and IV in the TNM classification) is poor, and its 5-year survival rate remains around 50%, despite much progress being made in multidisciplinary therapy such as combinations of chemo-radiotherapy and surgery [3]. The establishment of distinct prognostic factors and the development of novel treatments for advanced laryngeal cancer are important for the proper assessment of prognosis and for decision making about the most appropriate therapeutic regimen.

Cancer immunity plays an important role in the suppression of invasion and proliferation of solid tumors, but some types of cancer cells develop immune tolerance and can escape the T-cell mediated antitumor immune response [4, 5]. It is thus necessary to determine the precise status of the immune response in patients with advanced laryngeal cancer; doing so is likely to contribute to the development of distinct prognostic factors and/or novel treatments. In the present study, we focused on dendritic cells (DCs) in laryngeal cancer, which are generally considered to be central regulators of anticancer immune responses [6]. DCs are antigen-presenting cells that activate cytotoxic T-lymphocytes (CTLs) through the major histocompatibility complex class I and class II molecules [7]. DCs also directly activate B lymphocytes [8] and can activate innate immune cells such as natural killer cells and natural killer T cells [9, 10]. Thus, DCs are considered to play a central role in antitumor immunity.

CD1a is a transmembrane glycoprotein that is associated with the antigen presentation of DCs. In contrast to the S100 protein, which is usually expressed on both immature and mature DCs [11], CD1a is considered to be specifically expressed on immature DCs [12]. However, CD1a remains one of the most poorly understood molecules in terms of its functional roles, even though it was first described in 1982 [12]. Several reports have indicated that the infiltration of CD1a-positive (CD1a^+^) DCs into tumor tissue is associated with favorable clinical outcomes in carcinomas of the ovary [13], oral cavity [14, 15], thyroid [16] and gallbladder [17]. However, the roles of tumor-infiltrating CD1a^+^ DCs and their clinical impact on patients with laryngeal cancer remain to be elucidated. We conducted the present study to: (1) assess the status of tumor infiltrating CD1a^+^ DCs in patients with advanced laryngeal cancer; and (2) to clarify the relationships between CD1a^+^ DCs and clinicopathological characteristics including outcomes.

## Methods

### Patients

The initial enrollees were 656 patients with laryngeal cancer who were treated at Saga University Hospital between 1990 and 2016. Among them, we excluded neoadjuvant cases including those treated with chemotherapy with or without radiotherapy, because the microenvironment of tumors may be different between treatment naïve and neoadjuvant cases. The 93 patients who underwent a total laryngectomy as the initial treatment for laryngeal cancer were assessed; there were 33 cases at Stage II, 22 cases at Stage III and 38 cases at Stage IV, based on the TNM classification (8th ed.) [18]. We excluded Stage II cases because none of these patients died from cancer progression. Of the remaining 60 cases with Stage III and IV laryngeal cancers, 3 were excluded because of the unavailability of cancer tissue specimens. A final total of 57 total laryngectomy cases with Stage III or IV laryngeal cancer were retrospectively analyzed. Comprehensive informed consent for the use of resected tissue for research was obtained from all patients, and the study protocol was approved by the Ethics Committee of the Faculty of Medicine at Saga University (No. 2020–04-R-19).

### Immunohistochemistry

Sections (4-μm) of formalin-fixed paraffin-embedded specimens of representative cancer tissue from each patient’s case were used for immunohistochemistry (IHC). The primary antibodies used were CD1a (Clone010, Ready to use; Dako, Glostrup, Denmark), and CD8 (CloneC8/144B, Ready to use; Dako). IHC was performed using an Autostainer plus® automatic stainer (Dako). The Envision^+^® System (Dako) was employed as the secondary antibody. Specimens on slides were visualized after diaminobenzidine tetrahydrochloride staining, and nuclei were counterstained with hematoxylin.

### Assessment of DCs and CD8^+^ CTLs

**Fig. 1:**
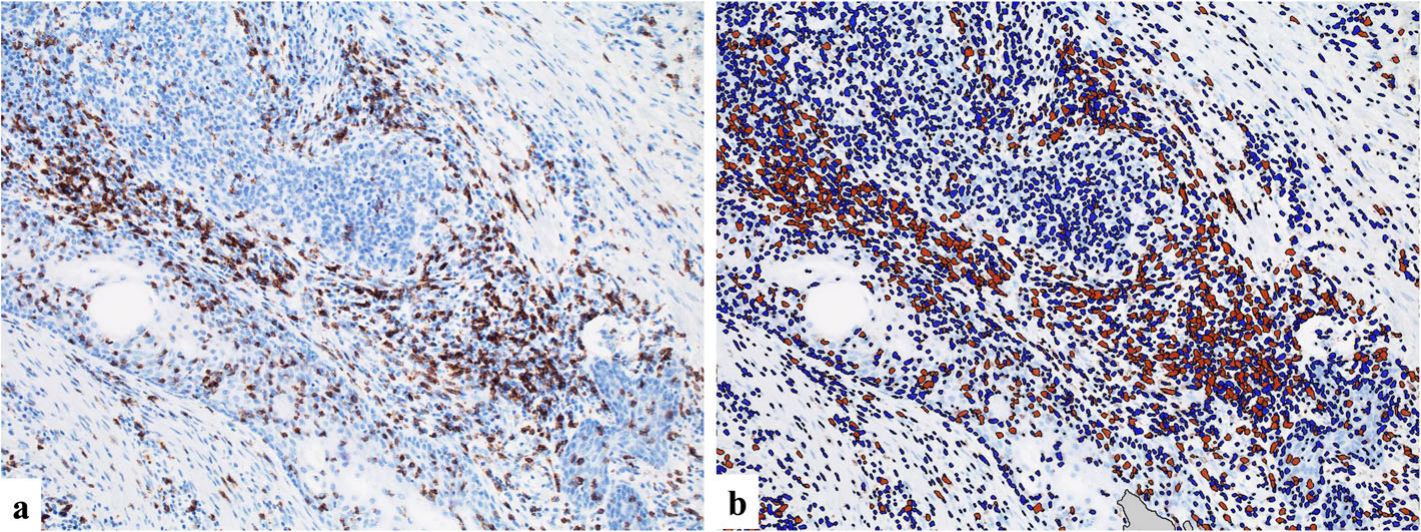
Representative analyzing images for CD8^+^ CTLs. **a** Digital image of IHC for CD8 (×200). **b** CD8^+^ CTLs automatically detected (*orange*) by image analysis software (Tissue Studio)

For the evaluation of CD1a^+^ and S100^+^ DCs, digital images of 3 hot spots of DC infiltration of tumor tissue were taken on a light microscope (× 100 magnification) for each patient. The number of DCs was counted in each digital image and the average number in the 3 digital images was calculated for each case. For the evaluation of CD8^+^ CTLs, digital photographs of 3 hot spots of CTL infiltration of tumor tissue were taken on a light microscope (× 200 magnification). The average number of tumor-infiltrating CD8^+^ CTLs in 3 digital images was automatically calculated by image analysis software (Tissue Studio, Definiens, Munchen, Germany). Representative analyzed images are shown in Fig. 1. We divided the patients into pairs of groups based on the median value for each assessment of CD1a^+^, S100^+^ and CD8^+^ cells.

### Statistical analysis

All statistical analyses were performed using JMP Pro 13.1.0 software (SAS Institute, Cary, NC, US). A Student’s *t*-test, Pearson’s chi-squared test and a linear regression analysis were used when appropriate for comparisons between 2 groups. Disease-specific survival (DSS) was defined as the period from surgery to cancer-related death or the last follow-up. Overall survival (OS) was defined as the period from surgery to death or the last follow-up. The maximum follow-up period in the study was 120 months, with a median follow-up time of 45.2 months. The survival curve was calculated by the Kaplan-Meier method, and a log-rank test was also conducted. Univariate and multivariate analyses were performed using a Cox proportional hazard model. Significant variables in the univariate analyses were selected for the multivariate analysis. *P*-values < 0.05 were considered to be significant.

## Results

### Clinicopathological features of the 57 patients with advanced laryngeal cancer

The clinicopathological features of the 57 patients with advanced laryngeal cancer are summarized in Table 1. Fifty-four patients (94.7%) were male and the remaining 3 (5.3%) were female. The mean age at the time of surgery was 68.4 years. The most frequent primary tumor site was the glottis (*n* = 35), followed by the supraglottis (*n* = 16) and the subglottis (*n* = 6). Regarding T stage, 4 cases (7.0%) were categorized as T2, 22 cases (38.6%) as T3, and 31 cases (54.4%) as T4. Thirty-seven cases (64.9%) had no lymph-node metastasis at the time of surgery. Only 1 patient had distant metastasis. Twenty cases (35.1%) were categorized as Stage III and the remaining 37 cases (64.9%) as Stage IV. The histology of all 57 cases was squamous cell carcinoma (SCC) and its histological differentiation was distributed as follows: well-differentiated SCC (*n* = 30), moderately differentiated SCC (*n* = 25) and poorly differentiated SCC (*n* = 2). Thirty-eight patients (66.6%) received adjuvant therapy as follows: 32 received chemotherapy (uracil/tegafur [*n* = 29], tegafur [*n* = 2], cisplatin+ 5-FU [*n* = 1]), 2 received radiotherapy and 4 received chemoradiotherapy; the chemotherapy regimens were: uracil/tegafur (*n* = 1), TS-1 (*n* = 1), cisplatin (*n* = 1) and nedaplatin (*n* = 1).

**Table 1:** Clinicopathological features of 57 patients with laryngeal cancer

Age, years (mean ± SD)	68.4 ± 8.8
Sex	
Male	54 (94.7%)
Female	3 (5.3%)
Smoking habit	
Never	5 (8.8%)
Ex	19 (33.3%)
Current	33 (57.9%)
Alcohol abuse	
(+)	35 (61.4%)
(−)	22 (38.6%)
Subsite	
Glottis	35 (61.4%)
Supraglottis	16 (28.1%)
Subglottis	6 (10.5%)
Histology^a^	
Well	30 (52.6%)
Mode	25 (43.9%)
Poor	2 (3.5%)
T stage	
T2	4 (7.0%)
T3	22 (38.6%)
T4	31 (54.4%)
N stage	
N0	37 (64.9%)
N1	5 (8.8%)
N2	14 (24.6%)
N3	1 (1.8%)
M stage	
M0	56 (98.2%)
M1	1 (1.8%)
Stage	
III	20 (35.1%)
IV	37 (64.9%)
Adjuvant therapy	
None	19 (33.3%)
Radiotherapy	2 (3.5%)
Chemotherapy	32 (56.1%)
Chemoradiotherapy	4 (7.0%)

^a^The histology of all cases was squamous cell carcinoma

### Assessment of CD1a^+^ DCs and their correlations with clinicopathological factors

**Fig. 2:**
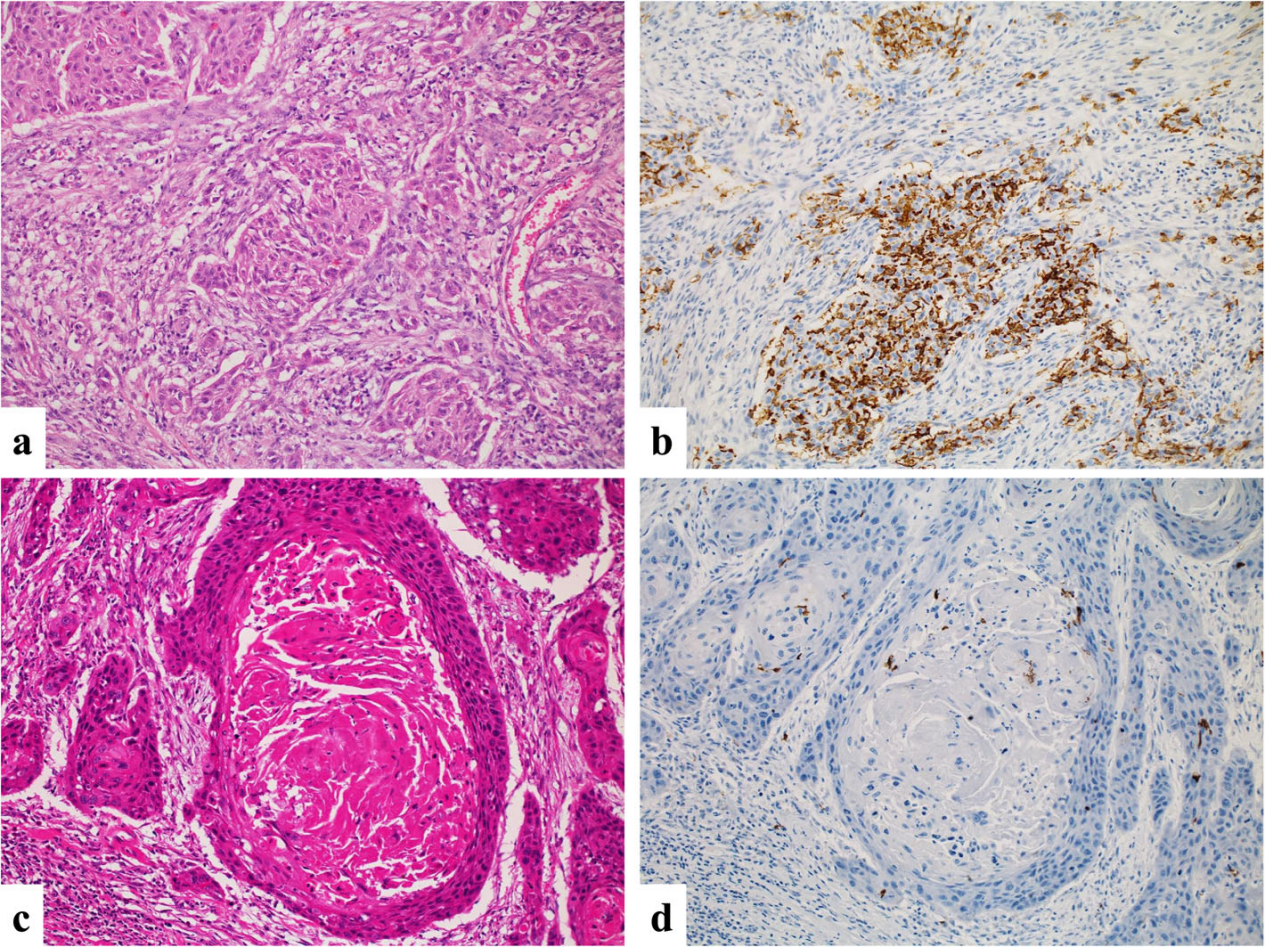
Representative histological images of CD1a^+^ DC infiltration into laryngeal squamous cell carcinoma. **a** Hematoxylin and eosin (HE)-stained image of a CD1a-high case (×200). **b** IHC of CD1a in a CD1a-high case (×200). Numerous CD1a^+^ DCs can be observed. **c** IHC of a CD1a-low case (× 200). **d** IHC of CD1a in a CD1a-low case (× 200). Few CD1a^+^ DCs are present

An infiltration of CD1a^+^ DCs was observed in all 57 cases, with various densities in areas adhering to or adjacent to tumor cells. The average number of tumor-infiltrating CD1a^+^ DCs was 47 (range 1.3–351). Using the median value (29) of the number of infiltrating CD1a^+^ DCs as the cut-off, we divided the cases into the CD1a-low group (*n* = 29) and the CD1a-high group (*n* = 28). Representative histological images from each group are shown in Fig. 2.

The relationships between CD1a^+^ DC infiltration and clinicopathological factors are summarized in Table 2. Details of CD1a^+^ DC and T- or N-stage (CD1a-high case no., CD1a-low case no.) were as following: T1 (2, 2), T3 (15, 7), T4 (12, 19), N0 (20, 17), N1 (4, 1), N2 (5, 9), N3 (0, 1). We divided the cases into T2/T3 vs T4, and N0 vs N1–3 for the statistical analyses because of the balance of case numbers. The CD1a-high group had significantly more advanced (T4 and Stage IV) cases (*P* = 0.045 and *P* = 0.038, respectively) than the CD1a-low group. No significant difference was observed between the CD1a-low and CD1a-high groups for other factors, i.e., age, sex, smoking, alcohol consumption, primary site, histological differentiation, T stage, N stage, M stage or adjuvant therapy.

**Table 2:** Clinicopathological features per CD1a^+^ DC infiltration

	CD1a Low (***n*** = 29)	CD1a High (***n*** = 28)	***P***-value
Age, years (mean ± SD)	68.9 ± 8.4	67.9 ± 9.3	0.682
Sex			
Male	28 (96.6%)	26 (92.9%)	0.532
Female	1 (3.4%)	2 (7.1%)	
Subsite			
Glottic	17 (58.6%)	18 (64.3%)	0.661
Supra/Sub	12 (41.4%)	10 (35.7%)	
Histology			
Well	16 (55.2%)	14 (50.0%)	0.696
Mode/poor	13 (44.8%)	14 (50.0%)	
T stage			
T2/T3	17 (58.6%)	9 (32.1%)	0.045
T4	12 (41.4%)	19 (67.9%)	
N stage			
N0	20 (69.0%)	17 (60.7%)	0.514
N1–3	9 (31.0%)	11 (39.3%)	
M stage (%)			
M0	28 (96.6%)	28 (100.0%)	0.321
M1	1 (3.4%)	0 (0.0%)	
Stage			
III	14 (48.3%)	6 (21.4%)	0.034
IV	15 (51.7%)	22 (78.6%)	
CD8^+^ CTLs	510.7 ± 582.6	616.7 ± 415.6	0.428
Adjuvant therapy			
(−)	8 (27.6%)	11 (39.3%)	0.349
(+)	21 (72.4%)	17 (60.7%)	

### Assessment of S100^+^ DCs and their correlations with clinicopathological factors

**Fig. 3:**
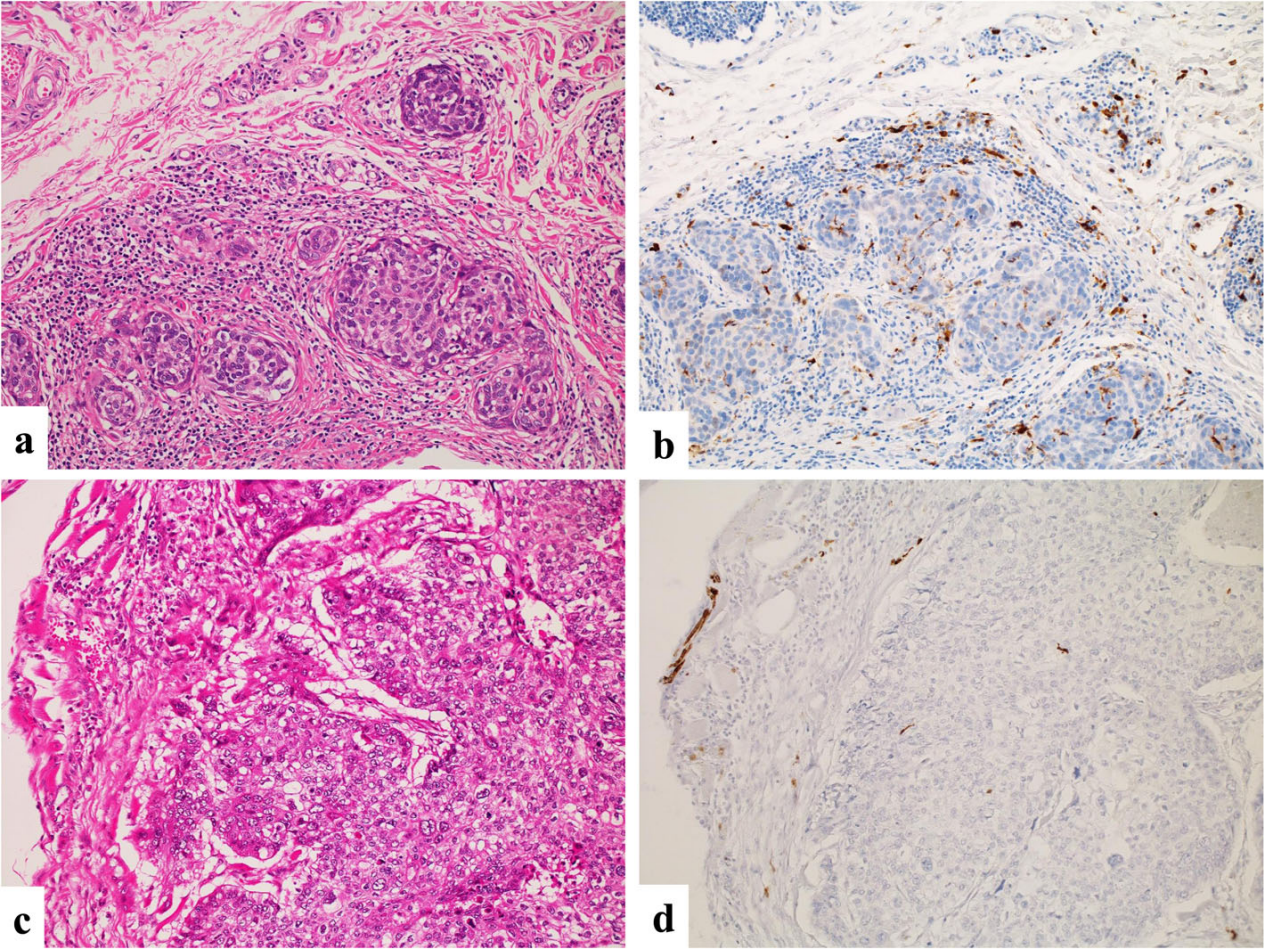
Representative histological images of S100^+^ DC infiltration in laryngeal squamous cell carcinoma. **a** A S100-high case (HE; × 200). **b** IHC of S100 in an S100-high case (× 200). **c** A S100-low case (HE; × 200). **d** IHC of S100 in a S100-low case (× 200)

Infiltration of S100^+^ DCs was observed in all 57 cases at various densities. The average number of tumor-infiltrating S100^+^ DCs was 69 (range 0.5–390). Using the median value (49) of S100^+^ DCs as the cut-off, we divided the cases into a S100-low group (*n* = 29) and a S100-high group (*n* = 28). Representative histological images from each group are shown in Fig. 3. The relationships between S100^+^ DC infiltration and clinicopathological factors are summarized in Table 3. Compared to the S100-low group, the S100-high group had significantly more cases of well-differentiated SCC (*P* = 0.012). No significant differences were found for other factors (age, sex, smoking, alcohol drinking, primary site, T stage, N stage, M stage or adjuvant therapy) between the S100-low and S100-high groups.

**Table 3:** Clinicopathological features per S100^+^ DC infiltration

	S100 Low (***n*** = 29)	S100 High (***n*** = 28)	***P***-value
Age, years (mean ± SD)	68.4 ± 10.0	68.4 ± 7.6	0.995
Sex (%)			
Male	28 (96.6%)	26 (92.9%)	0.532
Female	1 (3.4%)	2 (7.1%)	
Subsite			
Glottic	19 (65.5%)	16 (57.1%)	0.516
Supra/sub	10 (34.5%)	12 (42.9%)	
Histology			
Well	20 (69.0%)	10 (35.7%)	0.012
Mode/poor	9 (31.0%)	18 (64.3%)	
T stage (%)			
T2/T3	12 (41.4%)	14 (50.0%)	0.514
T4	17 (58.6%)	14 (50.0%)	
N stage (%)			
N0	22 (75.9%)	15 (53.6%)	0.078
N1–3	7 (24.1%)	13 (46.4%)	
M stage (%)			
M0	28 (96.6%)	28 (100.0%)	0.322
M1	1 (3.4%)	0 (0.0%)	
Stage			
III	11 (37.9%)	9 (32.1%)	0.647
IV	18 (62.1%)	19 (67.9%)	
CD8^+^ CTLs	499.2 ± 513.6	628.6 ± 498.2	0.541
Adjuvant therapy			
(−)	11 (37.9%)	8 (28.6%)	0.454
(+)	18 (62.1%)	20 (71.4%)	

### Assessment of CD8^+^ CTLs and their association with CD1a^+^ and S100^+^ DCs

**Fig. 4:**
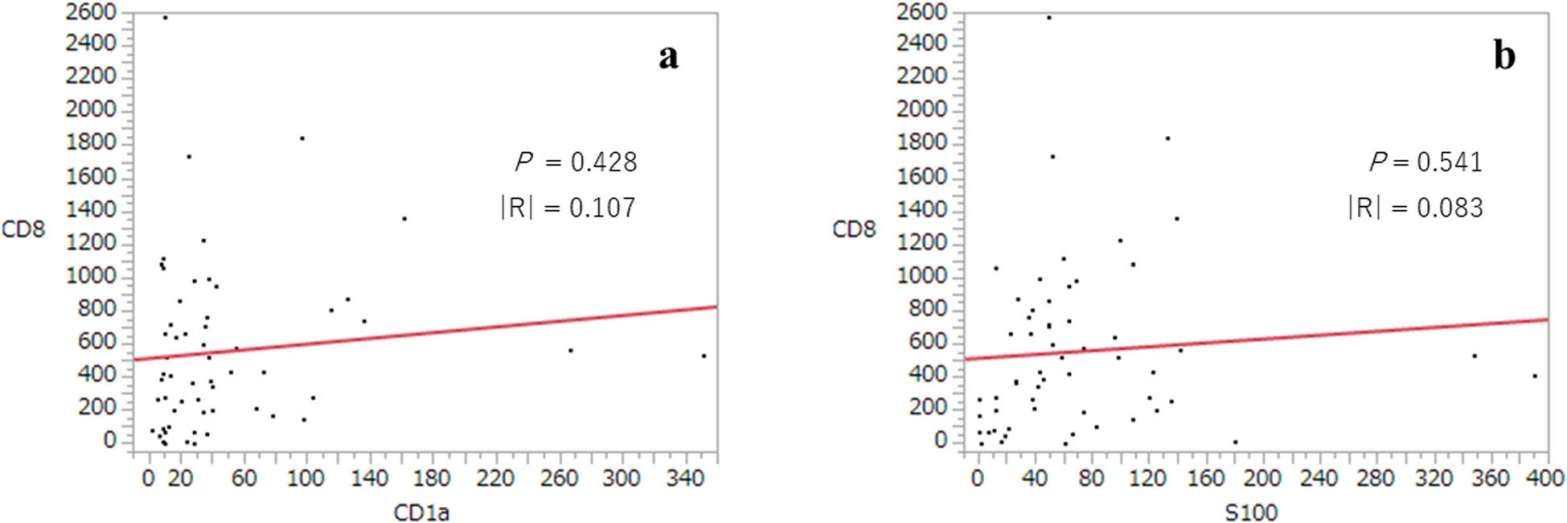
Linear regression analyses. **a** between CD1a^+^ DCs and CD8^+^ CTLs and **b** between S100^+^ DCs and CD8^+^ CTLs

The average number of tumor-infiltrating CD8^+^ CTLs was 563 (range 0–2, 580). The results of the linear regression analysis between CD1a^+^ DCs or S100^+^ DCs and CD8^+^ CTLs are illustrated in Fig. 4. No significant association were observed between tumor-infiltrating CD1a^+^ DCs and CD8^+^ CTLs, or between tumor-infiltrating S100^+^ DCs and CD8^+^ CTLs. We divided the cases into a CD8-low group (*n* = 28) and a CD8-high group (*n* = 29) using the median value of CD8^+^ CTLs (435) as the cut-off value. No significant differences in clinicopathological factors were observed between these groups.

### Kaplan-Meier survival curves according to the infiltration of DCs and CD8^+^ CTLs

**Fig. 5:**
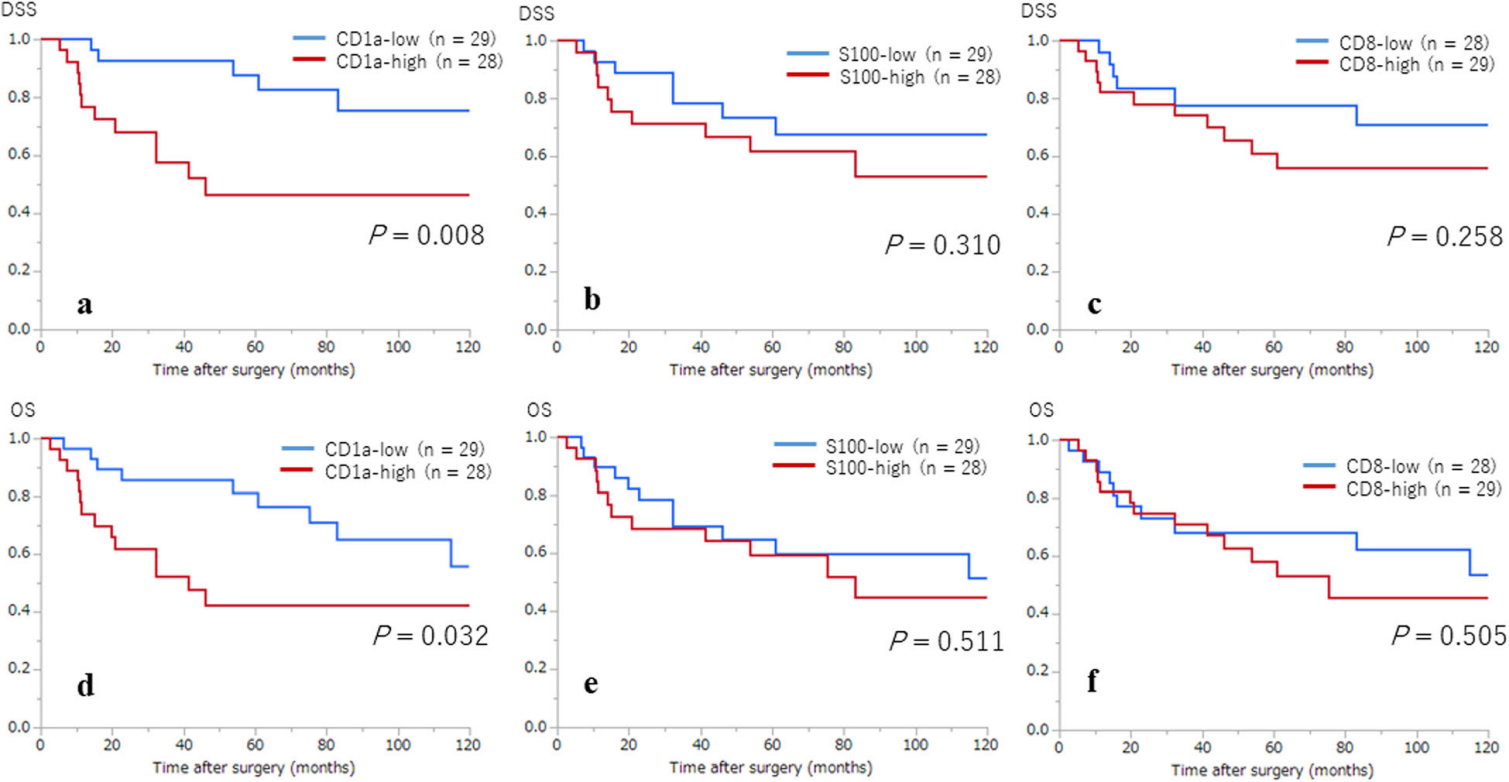
Kaplan-Meier survival curves according to CD1a, S100 and CD8 status in advanced laryngeal cancer. **a–c** Disease-specific survival (DSS) according to CD1a, S100, and CD8, respectively. **d–f** Overall survival (OS) according to CD1a, S100, and CD8, respectively. The number at risk is equal to the total number of patients because our cohort was restricted to advanced laryngeal cancer

The Kaplan-Meier survival curves, based on the status of CD1a^+^ DCs, S100^+^ DC and CD8^+^ CTLs, are shown in Fig. 5**.** The CD1a-low group had significantly better DSS and OS than the CD1a-high group (*P* = 0.008 and 0.032, respectively). No significant difference was found between the S100-low group and S100-high group (*P* = 0.310 and. *P* = 0.511) or between the CD8-low group and CD8-high group (*P* = 0.258 and *P* = 0.505) for both DSS and OS.

### Univariate and multivariate analyses for OS and DSS

The results of the univariate analyses for OS and DSS are summarized in Table 4. The factors significantly correlated with OS were tumor subsite, T stage, N stage and infiltration of CD1a^+^ DCs (*P* = 0.046, *P* = 0.008, *P* = 0.003, *P* = 0.035, respectively). The factors significantly correlated with DSS were T stage, N stage and infiltration of CD1a^+^ DCs (*P* = 0.000, *P* = 0.024, *P* = 0.009, respectively).

**Table 4:** Univariate analyses for disease specific-survival and overall survival (*n* = 57)

Characteristic	n	DSS		OS	
		HR (95% CI)	***P***-value	HR (95% CI)	***P***-value
Age			0.919		0.789
≤ 67 years	28	1		1	
> 67 years	29	1.05 (0.40–2.73)		1.12 (0.49–2.55)	
Sex			0.315		0.081
Female	3	1		1	
Male	54	0.29 (0.04–2.25)		0.20 (0.05–0.89)	
Subsite			0.149		0.046
Glottic	35	1		1	
Supra/sub	22	2.02 (0.78–5.25)		2.31 (1.01–5.28)	
Histology			0.575		0.937
Well	30	1		1	
Mode/poor	27	0.76 (0.29–2.00)		1.03 (0.45–2.35)	
T stage			0.000		0.008
T2/T3	26	1		1	
T4	31	8.93 (2.04–39.18)		3.24 (1.27–8.25)	
N stage			0.024		0.003
N0	37	1		1	
N1–3	20	3.05 (1.17–7.93)		3.58 (1.56–8.19)	
M stage			0.436		0.530
M0	56	1		1	
M1	1	2.50 (0.33–19.01)		2.04 (0.27–15.32)	
Adjuvant therapy			0.269		0.571
(−)	19	1		1	
(+)	38	1.83 (0.59–5.61)		1.29 (0.53–3.14)	
CD1a^+^ DCs			0.009		0.035
Low	43	1		1	
High	14	3.76 (1.31–10.74)		2.45 (1.05–5.70)	
S100^+^ DCs			0.416		0.672
Low	39	1		1	
High	18	1.49 (0.57–3.92)		1.19 (0.52–2.72)	
CD8^+^ CTLs			0.253		0.504
Low	33	1		1	
High	24	1.77 (0.65–4.84)		1.33 (0.58–3.06)	

*DS*S Disease-specific survival, *OS* Overall survival

The factors that were shown to be significant in the univariate analyses were further subjected to multivariate analysis (Table 5). The multivariate analysis for OS indicated that T stage, N stage and infiltration of CD1a^+^ DCs were each significantly associated with the patients’ OS (*P* = 0.026, *P* = 0.007, *P* = 0.013, respectively). The multivariate analysis for DSS indicated that T stage, N stage and infiltration of CD1a^+^ DCs were significantly associated with the patients’ DSS (*P* = 0.001, *P* = 0.021 and *P* = 0.009, respectively).

**Table 5:** Multivariate analyses for disease specific and overall survival (*n* = 57)

Type	Characteristic	HR (95%CI)	***P***-value
*DSS*	CD1a (high)	4.03 (1.32–12.29)	0.009
	T stage (T4)	8.48 (1.89–37.98)	0.001
	N stage (positive)	3.21 (1.21–8.57)	0.021
*OS*	CD1a (high)	3.14 (1.24–7.95)	0.013
	Subtype (glottic)	0.47 (0.19–1.13)	0.090
	T stage (T4)	2.78 (1.07–7.22)	0.026
	N stage (positive)	3.23 (1.38–7.54)	0.007

*DSS* Disease-specific survival, *OS* Overall survival

## Discussion

We investigated the infiltration of CD1a^+^ DCs and its association with clinicopathological factors in patients with advanced laryngeal cancer who underwent a total laryngectomy as an initial treatment. CD1a^+^ DCs infiltration was significantly associated with more advanced (T4 and Stage IV) cases. Unexpectedly, the CD1a-high group showed unfavorable clinical outcomes and it was an independent prognostic factor in multivariate analyses which involved TMN staging, whereas tumor-infiltrating S100^+^ DCs were not significantly associated with clinical outcomes. As S100^+^ DCs represent both mature and immature DCs, our results indicated that immature CD1a^+^ DCs likely have more influence on the prognosis than mature DCs. Similar results suggesting a significant effect on survival after immature CD1a^+^ DCs infiltration into tumor compared to mature DCs have been reported for several body organs [15–17].

It has been speculated that a higher density of CD1a^+^ DCs in tumor tissue correlates with favorable clinical outcomes, and several studies have reported that tumor-infiltrating CD1a^+^ DCs were associated with favorable clinical outcomes in carcinomas of various organs [13–17]. However, conflicting results have also been published. Hilly et al. [19] reported that higher CD1a^+^ DCs infiltration around a tumor was associated with a greater risk of recurrence in surgically treated cases of early squamous cell carcinoma of the tongue. Lundgren et al. [20] reported that a high density of infiltrating CD1a^+^ DCs was an unfavorable prognostic factor in a pancreato-biliary type of periampullary adenocarcinoma. One of the possible reasons for this discrepancy in findings are the widely varying methods used to evaluate CD1a^+^ DCs in previous reports. Taken together, the past and present findings show that the relationship between tumor-infiltrating CD1a^+^ DCs and clinical outcomes in patients with malignancies remains controversial.

Our literature search revealed 4 studies that investigated tumor-infiltrating DCs in laryngeal cancer, and 3 of these 4 studies investigated S100^+^ DCs only. Yilmaz et al. [21] and Gallo et al. [22] reported that a high density of tumor-infiltrating S100^+^ DCs was associated with favorable patient outcomes. However, Karakök et al. reported that the infiltration of S100^+^ DCs was not associated with survival, although it was significantly associated with the inflammatory response [23]. This result is consistent with our analysis of S100^+^ DCs. Only 1 of these 4 previous studies investigated CD1a^+^ DC infiltration in laryngeal cancer. Esteban et al. focused on CD1a (OKT6^+^) DCs and reported that the infiltration of CD1a^+^ DCs was not associated with survival, although it was significantly associated with lymphocyte infiltration [24]. Thus, the present study, we believe, is the first to report an association between poor surgical outcomes and tumor infiltrating CD1a^+^ DCs in laryngeal cancer.

It is generally considered that immature DCs capture the tumor antigen, mature and then present the antigen to naïve T cells, which induces a cellular immune response involving CD8^+^ CTLs [25]. In the present study, the number of CD8^+^ CTLs appeared to be greater in the CD1a^+^ DC-high group, although the result did not reach statistical significance. A possible explanation of the correlation of the adverse effect of CD1a^+^ DCs is that CD1a^+^ DCs have a specific but unknown function other than antigen presentation that accelerates the progression of cancer cells. It can also be hypothesized that the function of CD1a^+^ DCs in tumor tissue may differ according to the organ or histological cell type. As it is known that there are many subsets of DCs with unique and specific functions [26], these hypotheses seem plausible. However, the role(s) of CD1a^+^ DCs in cancer tissue remain to be unequivocally established, as do the reasons why the invasion of CD1a^+^ DCs is correlated with a poor prognosis of patients with advanced laryngeal cancer.

Recent studies have reported that high-CD8^+^ CTLs infiltration was associated with better prognosis of patients with laryngeal cancer [27, 28]. However, our study did not find a significant association between CD8^+^ CTLs infiltration and prognosis. Possible reasons for this discrepancy are different sample sizes and different selection criteria for patients. In contrast to previous research, the present study excluded neoadjuvant cases because the microenvironment of tumors may be different between treatment naïve and neoadjuvant cases. We considered treatment naïve cases were more suitable for this study because there is a paucity of knowledge about the role(s) of CD1a^+^ DCs in laryngeal cancer. Another possible reason is a difference in the assessment method for CD8^+^ CTLs. Although there is no consensus at present, further studies will contribute to the development of a unified method and the optimal cut-off assessment of CD8^+^ CTLs in laryngeal cancer.

It has been reported that head and neck SCC reduces the body’s immunocompetence in multiple ways [29]. A malfunction or decrease of plasmacytoid dendritic cells (pDCs) in tumor tissue is considered to be one of the causes of reduced immunocompetence because pDCs produce interferon (IFN), which plays an important role in antitumor immunity [30]. However, in contrast, without stimulation (e.g., by viruses), T cell derived CD40 ligand activates pDCs, and these activated pDCs support the functions of regulatory T cells and contribute to immunotolerance [31, 32]. O’Donnell et al. reported that an intratumoral increase of Langerin-positive immature DCs was significantly associated with vascular/lymphatic invasion and unfavorable survival in patients with oral SCC. They also found that the presence of CD123-positive pDCs was associated with a poor prognosis [33]. Thus, a malignancy’s microenvironment, which is induced and regulated by the immune system, is extremely complex and most of the functions and potential interactions among various subsets of DCs remain to be elucidated.

The limitations of the present study are its retrospective nature, the relatively small number of patients treated at a single center, and the long period required for enrollment. The treatment policy for neoadjuvant therapy is ununified and detailed information on the recurrence/metastatic lesion rates and treatment for these lesions are regrettably unavailable for our patient cohort. In addition, the immunohistochemical analyses were performed using only one representative section of cancer tissue, and therefore the entire tumor tissue was not evaluated.

## Conclusions

We have analyzed the association between the infiltration of CD1a^+^ DCs into cancer tissue and clinicopathological factors in patients with advanced laryngeal cancer. Our results demonstrated that CD1a^+^ DC infiltration was associated with a poor clinical outcome, and was an independent prognostic factor, revealed by multivariate analyses. The mechanism of CD1a^+^ DCs leading to unfavorable clinical outcomes remains to be clarified. We anticipate that further studies will validate our findings and ultimately elucidate the function(s) of tumor-infiltrating CD1a^+^ DCs in advanced laryngeal cancer.

## Data Availability

The datasets generated and/or analyzed during the current study are not publicly available due to the specifics of the patients’ informed consent and because the study’s ethics approval did not cover this issue, but they are available from the corresponding author on reasonable request.

## Abbreviations

CTL: Cytotoxic T-lymphocyte
DC: Dendritic cell
DSS: Disease-specific survival
IHC: Immunohistochemistry
OS: Overall survival
SCC: Squamous cell carcinoma

## References

[1] Cooper JS, Porter K, Mallin K, Hoffman HT, Weber RS, Ang KK, Gay EG, Langer CJ (2009). National Cancer Database report on cancer of the head and neck: 10-year update. Head Neck.

[2] Hori M, Matsuda T, Shibata A, Katanoda K, Sobue T, Nishimoto H (2015). Japan Cancer Surveillance Research Group. Cancer incidence and incidence rates in Japan in 2009: A study of 32 population-based cancer registries for the Monitoring of Cancer Incidence in Japan (MCIJ) project. Jpn J Clin Oncol.

[3] Matsuda T, Ajiki W, Marugame T, Ioka A, Tsukuma H, Sobue T, Research Group of Population-Based Cancer Registries of Japan (2011). Population-based survival of cancer patients diagnosed between 1993 and 1999 in Japan: a chronological and international comparative study. Jpn J Clin Oncol.

[4] Iwai Y, Ishida M, Tanaka Y, Okazaki T, Honjo T, Minato N (2002). Involvement of PD-L1 on tumor cells in the escape from host immune system and tumor immunotherapy by PD-L1 blockade. Proc Natl Acad Sci U S A.

[5] Rabinovich GA, Gabrilovich D, Sotomayor EM (2007). Immunosuppressive strategies that are mediated by tumor cells. Annu Rev Immunol.

[6] Ueno H, Klechevsky E, Morita R, Aspord C, Cao T, Matsui T, Di Pucchio T, Connolly J, Fay JW, Pascual V, Palucka AK, Banchereau J (2007). Dendritic cell subsets in health and disease. Immunol Rev.

[7] Banchereau J, Steinman RM (1998). Dendritic cells and the control of immunity. Nature..

[8] Jego G, Pascual V, Palucka AK, Banchereau J (2005). Dendritic cells control B cell growth and differentiation. Curr Dir Autoimmun.

[9] Fujii S, Shimizu K, Kronenberg M, Steinman RM (2002). Prolonged IFN-gamma-producing NKT response induced with alpha-galactosylceramide-loaded DCs. Nat Immunol.

[10] Lucas M, Schachterle W, Oberle K, Aichele P, Diefenbach A (2007). Dendritic cells prime natural killer cells by trans-presenting interleukin 15. Immunity..

[11] Donato R, Cannon BR, Sorci G, Riuzzi F, Hsu K, Weber DJ, Geczy CL (2013). Functions of S100 proteins. Curr Mol Med.

[12] Coventry B, Heinzel S (2004). CD1a in human cancers: a new role for an old molecule. Trends Immunol.

[13] Eisenthal A, Polyvkin N, Bramante-Schreiber L, Misonznik F, Hassner A, Lifschitz-Mercer B (2001). Expression of dendritic cells in ovarian tumors correlates with clinical outcome in patients with ovarian cancer. Hum Pathol.

[14] Goldman SA, Baker E, Weyant RJ, Clarke MR, Myers JN, Lotze MT (1998). Peritumoral CD1a-positive dendritic cells are associated with improved survival in patients with tongue carcinoma. Arch Otolaryngol Head Neck Surg..

[15] Jardim JF, Gondak R, Galvis MM, Pinto CAL, Kowalski LP (2018). A decreased peritumoral CD1a+ cell number predicts a worse prognosis in oral squamous cell carcinoma. Histopathology..

[16] Hilly O, Rath-Wolfson L, Koren R, Mizrachi A, Hamzany Y, Bachar G, Shpitzer T (2015). CD1a-positive dendritic cell density predicts disease-free survival in papillary thyroid carcinoma. Pathol Res Pract.

[17] Kai K, Tanaka T, Ide T, Kawaguchi A, Noshiro H, Aishima S (2021). Immunohistochemical analysis of the aggregation of CD1a-positive dendritic cells in resected specimens and its association with surgical outcomes for patients with gallbladder cancer. Transl Oncol.

[18] Brierley JD, Gospodarowicz MK, Wittekind C (2017). Union for International Cancer Control: TNM classification of malignant tumours.

[19] Hilly O, Strenov Y, Rath-Wolfson L, Hod R, Shkedy Y, Mizrachi A, Koren R, Shpitzer T (2016). The predictive value of dendritic cells in early squamous cell carcinoma of the tongue. Pathol Res Pract.

[20] Lundgren S, Karnevi E, Elebro J, Nodin B, Karlsson MCI, Eberhard J, Leandersson K, Jirström K (2017). The clinical importance of tumour-infiltrating macrophages and dendritic cells in periampullary adenocarcinoma differs by morphological subtype. J Transl Med.

[21] Yilmaz T, Gedikoglu G, Celik A, Onerci M, Turan E (2005). Prognostic significance of Langerhans cell infiltration in cancer of the larynx. Otolaryngol Head Neck Surg.

[22] Gallo O, Libonati GA, Gallina E, Fini-Storchi O, Giannini A, Urso C, Bondi R (1991). Langerhans cells related to prognosis in patients with laryngeal carcinoma. Arch Otolaryngol Head Neck Surg.

[23] Karakök M, Bayazit YA, Ucak R, Ozer E, Kanlikama M, Mumbuc S, Sari I (2003). Langerhans cell related inflammatory reaction in laryngeal squamous cell carcinoma. Auris Nasus Larynx.

[24] Esteban F, Ruiz-Cabello F, Gonzalez-Moles MA, Lopez-Gonzalez MA, Funez R, Redondo M (2012). Clinical significance of langerhans cells in squamous cell carcinoma of the larynx. J Oncol.

[25] Banchereau J, Palucka AK (2005). Dendritic cells as therapeutic vaccines against cancer. Nat Rev Immunol.

[26] Geissmann F, Manz MG, Jung S, Sieweke MH, Merad M, Ley K (2010). Development of monocytes, macrophages, and dendritic cells. Science.

[27] Chatzopoulos K, Kotoula V, Manoussou K, Markou K, Vlachtsis K, Angouridakis N, Nikolaou A, Vassilakopoulou M, Psyrri A, Fountzilas G (2020). Tumor infiltrating lymphocytes and CD8+ T cell subsets as prognostic markers in patients with surgically treated laryngeal squamous cell carcinoma. Head Neck Pathol.

[28] Hoesli R, Birkeland AC, Rosko AJ, Issa M, Chow KL, Michmerhuizen NL, Mann JE, Chinn SB, Shuman AG, Prince ME, Wolf GT, Bradford CR, McHugh JB, Brenner JC, Spector ME (2018). Proportion of CD4 and CD8 tumor infiltrating lymphocytes predicts survival in persistent/recurrent laryngeal squamous cell carcinoma. Oral Oncol.

[29] Young MR (2006). Protective mechanisms of head and neck squamous cell carcinomas from immune assault. Head Neck..

[30] Hartmann E, Wollenberg B, Rothenfusser S, Wagner M, Wellisch D, Mack B, Giese T, Gires O, Endres S, Hartmann G (2003). Identification and functional analysis of tumor-infiltrating plasmacytoid dendritic cells in head and neck cancer. Cancer Res.

[31] Gilliet M, Liu YJ (2002). Generation of human CD8 T regulatory cells by CD40 ligand-activated plasmacytoid dendritic cells. J Exp Med.

[32] Rissoan MC, Soumelis V, Kadowaki N, Grouard G, Briere F, de Waal MR, Liu YJ (1999). Reciprocal control of T helper cell and dendritic cell differentiation. Science..

[33] O'Donnell RK, Mick R, Feldman M, Hino S, Wang Y, Brose MS, Muschel RJ (2007). Distribution of dendritic cell subtypes in primary oral squamous cell carcinoma is inconsistent with a functional response. Cancer Lett.

